# Does the presence of neuropathic pain affect the outcomes of the interlaminar epidural steroid injection for cervical disc herniation? A prospective clinical study

**DOI:** 10.1097/MD.0000000000025012

**Published:** 2021-03-05

**Authors:** Canan Sanal-Toprak, Ekim Can Ozturk, Feyza Nur Yucel, Savas Sencan, Osman Hakan Gunduz

**Affiliations:** a Department of Physical Medicine and Rehabilitation; b Department of Physical Medicine and Rehabilitation, Pain Medicine Section, School of Medicine, Marmara University, Istanbul, Turkey.

**Keywords:** disc herniation, interlaminar epidural steroid injections, neck pain, neuropathic pain

## Abstract

Epidural steroid injections (ESI) are commonly performed for the treatment of chronic cervical disc herniation (CDH). Although they are considered to be effective for both nociceptive and neuropathic types of pain, there is a lack of data regarding the impact of neuropathic pain (NP) and nociceptive pain components on treatment outcomes. The aim of this study is to compare the effectiveness of interlaminar epidural steroid injection (ILESI) between patients with predominantly NP and nociceptive pain due to CDH.

Sixty five participants were initially included in the study and assessed by numeric rating scale (NRS), neck pain and disability scale (NPDS), short form-12 (SF-12), and self-reported Leeds assessment of neuropathic symptoms and signs (S-LANSS) pain scale at baseline and 1 month, 3 months, 6 months after ILESI.

All patients were evaluated at 1st month and 3rd month follow-up periods while 54 of patients achieved to complete 6th month follow-up. There were significant improvements in all outcome measures for all time periods when compared with the pre-intervention scores. At baseline 24 (36.9%) of patients had predominantly NP in accordance with S-LANSS pain scale. The ratio of NP predominant patients reduced to 7.6% at 1st month, 12.3% at 3rd month, and 12.9% at 6th month with a significant difference for each follow-up period when compared with the baseline. Although all NRS and NPDS scores at baseline were significantly higher in patients with NP, improvement was significant at all follow-up periods in both groups. Minimal clinically important change in NRS was observed in >75% of patients at 1st, 3rd, and 6th month in both groups.

The results of this study showed that NP is present in one-third of the patients suffering from neck and radiating arm pain due to CDH and cervical ILESI is an effective treatment approach for both neuropathic and nociceptive components of pain.

Clinical Trials Registration Number: NCT04235478

## 1 Introduction

Cervical disc herniation (CDH) is frequently associated with neck and/or upper limb pain. It may consist of both nociceptive and neuropathic components. Pain in the upper limb may be caused by either nociceptive referred pain or neuropathic radicular pain. Nociceptive referred pain is caused by a noxious stimulation of structures such as muscles, joints, ligaments, and intervertebral discs of the spine, which induces upper limb pain in addition to the axial pain. Radicular pain is a frequent neuropathic pain syndrome in the upper limb and caused by mechanical compression or chemical inflammation of nerve roots and ectopic discharges originating from an irritated dorsal ganglion.^[1]^ Neuropathic pain (NP) is defined as pain resulting from a lesion or disease affecting the somatosensory system.^[2]^ NP tends to be more severe than nociceptive pain with distinctive characteristics thus it requires different treatment strategies.^[3,4]^ In addition, it has been shown that NP is associated with a greater impairment in the physical, psychological, and social-related quality of life (QoL) with an increase in the treatment-related cost by 28% to 52% than the other forms of pain.^[4,5]^ In order to determine the appropriate treatment strategy and get better outcomes, it is of utmost importance to identify both nociceptive and neuropathic components of pain in CDH.

Epidural steroid injections (ESIs) are commonly performed procedures in the treatment of chronic CDH for long term pain relief.^[6]^ These injections can be administered via interlaminar or transforaminal routes in the cervical spine.^[7]^ The transforaminal approach is associated with an increased risk for severe, life-threatening neurological complications. Therefore, interlaminar approach is often chosen to deliver the drug to cervical epidural space in daily practice.^[8]^ Although the exact mechanism of action of ESIs has not been fully elucidated yet, they are considered to have some favorable effects on the nociceptive and neuropathic pain generating signal transmission.^[9]^ Identifying characteristics of patients who are most likely to benefit from ESIs is important to prevent performing this invasive procedure to patients who have low probability of significant pain relief. Previous studies evaluating the effectiveness of cervical ESI for CDH have shown beneficial effects,^[10,11]^ but there is limited data concerning nociceptive and neuropathic pain relief separately after cervical ESI. In an earlier study which aimed to define the clinical characteristics of patients who might have better outcomes after cervical ESI, it has been reported that patients with radicular symptoms and signs had more pain relief when compared with those with axial neck pain.^[12]^ In a recent study, Lee and Lee^[13]^ also analyzed both neck pain and radiating upper limb pain reduction after cervical ESI, however the comparison of the outcomes between patients with predominantly NP and those with nociceptive pain has not been previously studied.

The aim of this study is to evaluate the effect of cervical interlaminar ESI (ILESI) on NP components of patients and to compare the outcome measures between patients with predominantly NP and nociceptive pain in order to determine the pain characteristic of patients who benefit more after cervical ILESI.

## 2 Materials and methods

### 2.1 Study design

Patients between 18 and 75 years old having neck and radiating arm pain for at least 3 months, diagnosed with CDH by clinical examination and magnetic resonance imaging (MRI) findings were recruited in this prospectively designed study. All participants were unresponsive to physical therapy modalities and medical treatment including gabapentinoids, nonsteroidal anti-inflammatory drugs, and antidepressants. After recording a numeric rating scale (NRS) score of 4 or above, ILESIs were performed at Pain Medicine division of Marmara University School of Medicine between December 2017 and December 2019. Approval was taken from the ethics committee for the protocol (Ethics committee number: 2017/119) and it was registered in clinicaltrials.gov (Identifier: NCT04235478). Both written and verbal consents were obtained before the procedure. The exclusion criteria were having any other musculoskeletal disorder that may be related with patients’ neck and arm pain, systematic or local infection, bleeding diathesis, allergy to contrast substances or local anesthetic agents, presence of systematic inflammatory disease, malignancy or neurological illness, entrapment neuropathies of upper limb, myelopathy on MRI, history of cervical ESI in the last 3 months, and neck surgery. The participants were evaluated 4 times in total; before the intervention, 1 month, 3 months, and 6 months after the intervention. Neck/shoulder, arm, and night pain scores, disability, QOL, and neuropathic pain were assessed using NRS, neck pain and disability scale (NPDS), short form-12 (SF-12), and self-reported Leeds assessment of neuropathic symptoms and signs (S-LANSS) pain scale, respectively. Patients were divided into 2 groups with respect to their initial S-LANSS score. A score of below 12 was interpreted as a sign of having nociceptive pain, those with higher scores were accepted to have predominantly NP.

### 2.2 Outcomes measures

#### 2.2.1 Self-reported Leeds assessment of neuropathic symptoms and signs (S-LANSS):

It is a 7-item self-reported scale developed to screen the presence of NP.^[14]^ There are 5 items regarding pain symptoms and 2 items involving self-administered sensory tests for the evaluation of allodynia and decreased sensation to pinprick. Responses to each item are yes or no and all items have specific weighted values. The score ranges from 0 to 24 and a score of ≥12 suggests the presence of NP. It has been demonstrated that Turkish version of S-LANSS is reliable and valid to identify the neuropathic pain.^[15]^

NRS: It is a numeric scale with 11 point which ranges from 0 (no pain) to 10 (worst pain). It is a simple and widespread used scale for rating pain intensity. A minimal clinically important change (MCIC) scores for both neck pain and referred pain have been reported to be 1.5 for NRS.^[16]^

#### 2.2.2 Neck pain and disability scale (NPDS):

This scale is a functional assessment questionnaire specifically developed for patients with neck pain by Wheeler et al^[17]^ and validated in Turkish in 2004 by Biçer et al.^[18]^ The questions investigate the intensity of neck pain and its relationship with emotional factors and vocational, recreational, social, and functional aspects of living. A 10-cm visual analog scale (VAS) is used for each question. Scoring for each question ranges from 0 to 5 and the total NPDS score varies from 0 to 100 points. Higher scores indicate severe disability in patients.

SF-12: This is one of the most widely used instruments to evaluate self-reported health related QOL. It is a shortened version of the SF-36 and covers the same 8 health domains, which reproduces 2 summary scores; mental component summary (MCS-12), and physical component summary (PCS-12). The scores range from 0 to 100 where higher scores indicated better QOL.

### 2.3 Injection technique

The procedure was carried by a pain physician having 10 years of experience in fluoroscopy guided interventions. Firstly, the patient was given a prone position and local anesthesia was applied with 3 cm^3^ of 2% prilocaine under sterilized conditions. After localizing C7–T1 interspace in anteroposterior view, the C-arm was adjusted to a contralateral oblique angle and under intermittent fluoroscopic imaging an 18G Touhy needle was advanced through right/left parasagittal part of C7–T1 interlaminar space. The loss of resistance technique was utilized to confirm that the needle was in the epidural space. A contrast medium was given to check non-vascularity and epidural spread prior to an injection of a mixture of 80 mg triamcinolone acetonide, 1 cm^3^ 2% lidocaine hydrochloride, and 2 cm^3^ 0.9% saline. The patients were discharged after 2 hours of observation period to be evaluated at 1st month follow-up.^[7]^

### 2.4 Statistical analyses

Statistical analyses were performed using the SPSS version 20.0 software (IBM Corp., Armonk, NY). Continuous variables were expressed in mean (standard deviation) and median (interquartile range), categorical variables were expressed in number and frequency. The chi-square and Fisher exact were used to compare categorical variables, where applicable. The Shapiro-Wilk test was used to analyze normal distribution of quantitative data. For the comparison of non-normally distributed data, the Mann–Whitney *U* was performed while the independent *t* test was used to compare normally distributed data. The Friedman test and Cochran *Q* test were conducted to determine changes over time with treatment for non-normally distributed data and repeated measures ANOVA was used for normally distributed data. Wilcoxon signed test, McNemar tests, and paired sample t-test were performed for pairwise comparisons with a Bonferroni correction for multiple comparisons. For Bonferroni correction, statistical significance was accepted at *P*-value <.0125 level, otherwise a *P*-value <.05 was considered statistically significant with 95% confidence intervals.

## 3 Results

A total of 65 patients were recruited to the study. While patients were fully compliant with follow-ups until 3rd month, 11 patients did not admit to 6th month follow-up: 6 from nociceptive pain group, 5 from NP group. ILESI procedures were performed at C7–T1 level in all subjects, 4 of them had vasovagal reaction after the intervention, no major complications were observed. There were no significant differences in sociodemograpic and clinical characteristics of patients such as sex, age, body mass index and symptom duration between 2 groups (Table 1).

**Table 1:** Neuropathic and nociceptive pain according to sociodemograpic and clinical characteristics of patients.

	Total cohort	Neuropathic pain	Nociceptive pain	*P* value
Age, y	51.3 (11.67)	52.5 (10.5)	50.6 (12.4)	.542^∗^
BMI, kg/m^2^	27.7 (4.21)	27.7 (3.8)	27.6 (4.5)	.971^∗^
Symptom duration, mo	12 (3.5–24)	12 (3.25–12)	10 (3.5–18)	.735^†^
Sex				.055^‡^
Male	29 (44.6)	7 (29.2)	22 (53.7)	
Female	36 (55.4)	17 (70.8)	19 (46.3)	

Regarding the analysis of all patients, NRS, S-LANSS pain scale, NPDS scores at all follow-up points were significantly lower than those at baseline. Similarly, PCS-12 and MCS-12 follow-up scores (except MCS-12 score at 6th month) were significantly higher, referring to a better quality of life (Tables 2 and 3). The number of patients with predominantly neuropathic pain assessed with the S-LANSS pain scale was significantly decreased at all follow-up points when compared with baseline (Table 4).

**Table 2:** Pre- and post-procedural NRS scores for neck/shoulder, arm, and night pain.

	NRS-neck/shoulder	NRS-arm	NRS-night
	Median (IQR)	Median (IQR)	Median (IQR)
Pre	7.0 (6–8)	8.0 (6.5–8)	7.0 (6–8)
1 month	3.0 (1–4)	2.0 (0–4)	2.0 (0–4)
3 months	4.0 (1–5)	3.0 (1–5)	3.0 (1–5)
6 months	3.0 (1–4.25)	3.0 (0.75–5.25)	3.0 (0.75–5)
*P* value	**<.001**^∗^	**<.001**^∗^	**.001**^∗^
Pre versus 1 month	**<.001**^†^	**<.001**^†^	**<.001**^†^
Pre versus 3 months	**<.001**^†^	**<.001**^†^	**<.001**^†^
Pre versus 6 months	**<.001**^†^	**<.001**^†^	**<.001**^†^

**Table 3:** Pre- and post-procedural NPDS, PCS-12, MCS-12 scores of patients.

	NPDS	PCS-12	MCS-12
	Mean (SD)	Mean (SD)	Mean (SD)
Pre	67.1 (17.8)	34.0 (7.4)	39.2 (10.9)
1 month	43.0 (22.8)	38.1 (10.2)	45.3 (9.7)
3 months	42.6 (23.9)	39.0 (9.3)	43.8 (8.8)
6 months	40.0 (27.0)	40.5 (10.7)	42.1 (10.7)
*P* value	**<.001**^∗^	**<.001**^∗^	**.003**^∗^
Pre versus 1 month	**<.001**^†^	**.002**^†^	**<.001**^†^
Pre versus 3 months	**<.001**^†^	**<.001**^†^	**.005**^†^
Pre versus 6 month	**<.001**^†^	**<.001**^†^	.032^†^

**Table 4:** Pre- and post-procedural S-LANSS pain scale scores and number of patients with predominantly NP.

	S-LANSS score	Patients with NP
	Median (IQR)	n (%)
Pre	10 (5–13.5)	24 (36.9)
1 month	3.0 (0–9.5)	5 (7.6)
3 months	3.0 (0–10)	8 (12.3)
6 months	3.0 (0–9.25)	7 (12.9)
*P* value	**<.001**^‡^	**<.001**^§^
Pre versus 1 month	**<.001**^∗^	**<.001**^†^
Pre versus 3 months	**<.001**^∗^	**<.001**^†^
Pre versus 6 months	**<.001**^∗^	**.004**^†^

The initial NRS scores for neck/shoulder pain, for arm and night pain were significantly higher in patients with NP. Post-intervention NRS scores for arm pain at 1st and 3rd month and for night pain at 3rd month were significantly higher in the NP predominant patients. All NRS scores were significantly reduced at all follow-up points when compared with baseline in both groups (Table 5). MCIC for all NRS scores was observed in >75% of patients at 1st, 3rd, and 6th month in both groups and no significant differences were found between 2 groups (Table 6). The initial NPDS score was significantly higher in the NP predominant patients and NPDS scores were significantly reduced at all follow-up points in both groups. There were no differences in SF-12 sub-scores between 2 groups at any time points (Table 7).

**Table 5:** NRS scores for neck/shoulder, arm, and night pain according to neuropathic component.

	Neuropathic pain	Nociceptive pain	
NRS-neck/shoulder	Median (IQR)	Median (IQR)	*P* value
Pre	8.0 (7.25–9)	7.0 (6–8)	**.027**^∗^
1 month	3.5 (1–5)	3.0 (1–4)	.441^∗^
3 months	4.0 (1–5.75)	3.0 (1–4)	.159^∗^
6 months	4.0 (1–7)	3.0 (1–4)	.539^∗^
*P* value	**<.001**^‡^	**<.001**^‡^	
Pre versus 1 month	**<.001**^†^	**<.001**^†^	
Pre versus 3 months	**<.001**^†^	**<.001**^†^	
Pre versus 6 months	**<.001**^†^	**<.001**^†^	
NRS-arm			
Pre	8.0 (7.25–10)	7.0 (6–8)	**.004**^∗^
1 month	4.0 (1.25–6.75)	2.0 (0–3)	**.009**^∗^
3 months	5.0 (2–6.75)	3.0 (1–4)	**.013**^∗^
6 months	4.0 (1–7)	3.0 (0–4)	.152^∗^
*P* value	**<.001**^‡^	**<.001**^‡^	
Pre versus 1 month	**<.001**^†^	**<.001**^†^	
Pre versus 3 months	**<.001**^†^	**<.001**^†^	
Pre versus 6 months	**<.001**^†^	**<.001**^†^	
NRS-night			
Pre	8.0 (7.25–9.75)	7.0 (5–8)	**.003**^∗^
1 month	3.5 (1–5)	2.0 (0–3–5)	.069^∗^
3 months	4.5 (2–6.75	3.0 (0–4)	**.041**^∗^
6 months	4.0 (1–7)	3.0 (0–4)	.308^∗^
*P* value	**<.001**^‡^	**<.001**^‡^	
Pre versus 1 month	**<.001**^†^	**<.001**^†^	
Pre versus 3 months	**<.001**^†^	**<.001**^†^	
Pre versus 6 months	**<.001**^†^	**<.001**^†^	

**Table 6:** Patients with minimal clinically important change of NRS scores.

	Total cohort	Neuropathic pain	Nociceptive pain	
NRS-neck/shoulder	n (%)	n (%)	n (%)	*P*
Pre versus 1 month	55 (84.6)	19 (79.2)	36 (87.8)	.479^†^
Pre versus 3 months	51 (78.5)	18 (75.0)	33 (80.5)	.603^∗^
Pre versus 6 months	44 (81.5)	15 (78.9)	29 (82.9)	.728^†^
NRS-arm				
Pre versus 1 month	55 (84.6)	18 (75.0)	37 (90.2)	.154^†^
Pre versus 3 months	54 (83.5)	18 (75.0)	36 (87.8)	.304^†^
Pre versus 6 months	44 (81.5)	16 (80.0)	28 (84.2)	1.000^†^
NRS-night				
Pre versus 1 month	48 (81.3)	18 (78.3)	30 (83.3)	.736^†^
Pre versus 3 months	47 (79.6)	18 (78.3)	29 (80.6)	1.000^†^
Pre versus 6 months	41 (85.4)	15 (83.3)	15 (83.3)	1.000^†^

**Table 7:** NPDS, MCS-12, and PCS-12 scores according to neuropathic component.

	Neuropathic pain	Nociceptive pain	
NPDS	Mean (SD)	Mean (SD)	*P* value
Pre	73.9 (18.0)	63.0 (16.6)	**.016**^∗^
1 month	48.0 (24.0)	40.1 (21.8)	.182^∗^
3 months	49.0 (26.3)	38.9 (21.8)	.100^∗^
6 months	49.1 (33.0)	35.1 (22.2)	.068^∗^
*P* value	**<.001**^‡^	**<.001**^‡^	
Pre versus 1 month	**<.001**^†^	**<.001**^†^	
Pre versus 3 months	**<.001**^†^	**<.001**^†^	
Pre versus 6 months	**.004**^†^	**<.001**^†^	
MCS-12			
Pre	38.8 (13.1)	39.4 (9.6)	.842^∗^
1 month	46.8 (10.2)	44.4 (9.4)	.328^∗^
3 months	44.8 (8.7)	43.2 (8.9)	.489^∗^
6 months	40.5 (11.6)	43.0 (10.2)	.399^∗^
*P* value	**<.009**^‡^	<.163^‡^	
Pre versus 1 month	**.003**^†^		
Pre versus 3 months	.039^†^		
Pre versus 6 months	.245^†^		
PCS-12			
Pre	31.7 (8.5)	35.4 (6.4)	.055^∗^
1 month	35.9 (11.1)	39.4 (9.5)	.184^∗^
3 months	38.2 (10.5)	39.4 (8.7)	.608^∗^
6 months	39.7 (12.7)	41.0 (9.6)	.679^∗^
*P* value	**<.002**^‡^	**<.007**^‡^	
Pre versus 1 month	.075^†^	.016^†^	
Pre versus 3 months	**.004**^†^	.015^†^	
Pre versus 6 months	.024^†^	**.007**^†^	

## 4 Discussion

In the present study, the effects of ILESI on NP in patients with CDH were evaluated and the outcomes of patients with predominantly NP and nociceptive pain were compared. Herniated nucleus pulposus induces inflammation of the affected dorsal root ganglion and spinal nerves resulting in ectopic firing which is associated with increased glial activity in the spinal cord and the release of pain modulation agents.^[19]^ This cascade plays a role in development and maintenance of central sensitization-related NP.^[19]^ The current study showed that 24 of 65 patients (36.9%) had NP symptoms and signs in accordance with the S-LANSS pain scale at baseline, indorsing the role of these pathophysiological mechanisms in this condition. In a previous study which evaluated the presence of NP in patients with unilateral cervical radiculopathy, 30% of patients demonstrated a NP component according to the PainDETECT questionnaire.^[20]^ In the current study all CDH patients with neck and radiating arm pain were included. The S-LANSS pain scale has a high sensitivity (83%) and specificity (87%) for the diagnosis of NP and has been widely used for investigating the effectiveness of different treatment modalities in NP.^[14,21]^ Although there is no study in patients with CDH or cervical radiculopathy, it is an accepted questionnaire to depict pain with predominantly neuropathic origin and even offered as a diagnostic tool in lumbar radiculopathy.^[22]^

The ratio of patients with predominantly NP decreased from 36.9% to 7.6% at 1st month, 12.3% at 3rd month, and 12.9% at 6th month after ILESI, indicating a statistically significant difference at all time points. In addition, significant improvements were also observed in the S-LANSS, NRS, SF-12, and NPDS scores at all post-intervention time points, which are compatible with previous studies showing reduced pain and disability and advanced QoL after ILESI in patients with CDH.^[6,10,23]^ In the literature, although there is no data about the effects of ILESI on NP components of CDH, there are studies showing the effectiveness of ESIs on NP in patients with low back pain.^[24,25]^ The ESI exerts its anti-inflammatory effects on NP by reducing cytokine and chemokines, reducing or inhibiting neuroglial activation, inhibiting nociceptive C-fiber transmission and ectopic neuronal discharge and has stabilization effects by alleviating inflammation of the spinal nerves and epidural zone.^[19,26]^ Compared with those with nociceptive pain, the initial NRS and NPDS scores were significantly higher in the NP predominant patients. The NRS-arm at 1st and 3rd month and the NRS-night scores at 3rd month were also found to be higher in the same group. The intensiveness of NRS scores and NPDS scores in NP group indicate that NP predominant patients experience severe pain and disability than those with nociceptive pain, consistent with previous studies.^[27]^ The relatively poor treatment outcomes among the NP predominant patients compared with those with nociceptive pain can be attributed to the structural alteration of the synapses in the cornu of the spinal cord, interneurons, and glial cells which are involved in the development and chronicity of NP, and glial activation-induced central sensitization.^[28]^ In other respects, the fact that the initial pain scores in patients with NP were higher than those with nociceptive pain may be the reason for the difference between the 2 groups during follow-ups. On the other hand, the ratio of patients with MCIC were similar between groups at any time points which suggests that ILESI is effective in both nociceptive and neuropathic components of pain and the NP symptoms and signs before the intervention do not adversely affect the treatment outcomes.

Lack of a control group in consequence of ethical considerations and a relatively short follow-up period are the main limitations of this study. Radiologic assessment of the groups regarding nerve root compression grading, which might affect outcomes after ILESI was also omitted. Furthermore, S-LANSS scale might have missed some of the NP predominant cases. Although it is a useful tool to establish the presence of NP, about 20% to 30% of the patients with NP can be overlooked.^[29]^ In addition to S-LANSS scale, performing electroneuromyography for the diagnosis of an underlying radiculopathy which is the main cause of NP in patients with CDH could have increased the strength of the study. Nevertheless, the main strengths of the present study are its prospective design and novel findings on the effects of ILESI on NP in patients with CDH.

In conclusion, the results of this study showed that NP is predominant in about one-third of the patients suffering from neck and radiating arm pain due to CDH. These patients have higher neck, arm and night pain and disability scores when compared with those with nociceptive pain. Cervical ILESI is an effective and safe approach for the treatment of CDH, besides alleviating both nociceptive and neuropathic components of pain, it also reduces disability. In addition, the response to the ILESI of patients with NP is comparable to those with nociceptive pain, despite the presence of initial higher pain and disability scores.

## Author contributions

**Conceptualization:** Canan Sanal-Toprak, Savas Sencan, Osman Hakan Gunduz.

**Data curation:** Ekim Can Ozturk, Feyza Nur Yucel, Savas Sencan.

**Formal analysis:** Ekim Can Ozturk, Feyza Nur Yucel, Savas Sencan.

**Investigation:** Canan Sanal-Toprak, Ekim Can Ozturk, Feyza Nur Yucel, Savas Sencan.

**Methodology:** Ekim Can Ozturk, Feyza Nur Yücel, Savas Sencan.

**Supervision:** Canan Sanal-Toprak, Savas Sencan, Osman Hakan Gunduz.

**Writing – original draft:** Ekim Can Ozturk, Feyza Nur Yucel, Savas Sencan.

**Writing – review & editing:** Canan Sanal-Toprak, Savas Sencan, Osman Hakan Gunduz.
